# Methotrexate Disposition, Anti-Folate Activity, and Metabolomic Profiling to Identify Molecular Markers of Disease Activity and Drug Response in the Collagen-Induced Arthritis Mouse Model

**DOI:** 10.3390/metabo12010024

**Published:** 2021-12-28

**Authors:** Yezan M. Salamoun, Kishore Polireddy, Yu Kyoung Cho, Matthew R. Medcalf, Ryan S. Funk

**Affiliations:** 1 Department of Pharmacy Practice, University of Kansas Medical Center, Kansas City, KS 66160, USA; kishore414@gmail.com (K.P.); nadoangel007@gmail.com (Y.K.C.); mmedcalf@ku.edu (M.R.M.); 2 Department of Pharmacology, Toxicology and Therapeutics, University of Kansas Medical Center, Kansas City, KS 66160, USA

**Keywords:** metabolomics, rheumatoid arthritis, methotrexate, biomarkers, plasma metabolome, collagen-induced arthritis, folates

## Abstract

Methotrexate (MTX) is widely used in the treatment of autoimmune arthritis but is limited by its unpredictable and variable response profile. Currently, no biomarkers exist to predict or monitor early therapeutic responses to MTX. Using a collagen-induced arthritis (CIA) mouse model, this study aimed to identify biochemical pathways and biomarkers associated with MTX efficacy in autoimmune arthritis. Following arthritis disease induction, DBA/1J mice were treated with subcutaneous MTX (20 mg/kg/week) and disease activity was assessed based on disease activity scores (DAS) and paw volume (PV) measurements. Red blood cell (RBC) and plasma samples were collected at the end of the study and were assessed for folate and MTX content. Plasma samples were analyzed by semitargeted global metabolomic profiling and analyzed by univariate and multivariate analysis. Treatment with MTX was associated with significant reductions in disease activity based on both DAS (*p* = 0.0006) and PV (*p* = 0.0006). MTX therapy resulted in significant reductions in 5-methyltetrahydrofolate (5mTHF) levels in plasma (*p* = 0.02) and RBCs (*p* = 0.001). Reductions in both RBC and plasma 5mTHF were associated with lower DAS (*p* = 0.0007, *p* = 0.01, respectively) and PV (*p* = 0.001, *p* = 0.005, respectively). Increases in RBC MTX were associated with lower DAS (*p* = 0.003) but not PV (*p* = 0.23). Metabolomic analysis identified N-methylisoleucine (NMI) and quinolone as metabolites significantly altered in disease mice, which were corrected towards healthy control levels in mice treated with MTX. Reductions in plasma NMI were associated with lower DAS (*p* = 0.0002) and PV (*p* = 9.5 × 10^−6^). Increases in plasma quinolone were associated with lower DAS (*p* = 0.02) and PV (*p* = 0.01). Receiver-operating characteristic curve analysis identified plasma NMI (AUC = 1.00, *p* = 2.4 × 10^−8^), RBC 5mTHF (AUC = 0.99, *p* = 2.4 × 10^−5^), and plasma quinolone (AUC = 0.89, *p* = 0.01) as top discriminating metabolites of MTX treatment. Our data support a relationship between MTX efficacy and its effect on circulating folates and identified 5mTHF, NMI, and quinolone as potential therapeutic biomarkers of disease activity and MTX response in the CIA mouse model of autoimmune arthritis.

## 1. Introduction

Methotrexate (MTX) is a disease-modifying antirheumatic drug (DMARD) widely used in the treatment of rheumatoid arthritis (RA) and other autoimmune conditions [1,2]. However, clinical response to MTX therapy is variable and unpredictable, with treatment failure occurring in up to one-third of patients [3]. Furthermore, MTX therapy is characterized by a delayed onset of action, requiring up to 6 months to achieve maximal therapeutic response [1,3,4]. While early disease control has been shown to lead to improved long-term outcomes, progression of RA is associated with reduced life expectancy and chronic disability [5]. Hence early and effective drug therapy has become an increasingly important therapeutic goal. To date, there remains no established pretreatment or early treatment clinical biomarkers to stratify RA patients based on their likelihood to adequately respond to MTX [4]. As a result, the focus of this work is to use an autoimmune arthritis mouse model to identify molecular biomarkers and biochemical pathways associated with MTX response in search of potential biomarkers of MTX efficacy in RA.

Although MTX is widely used as a first line agent in the treatment of rheumatic diseases, the pharmacologic basis for its efficacy in RA remains unknown, although various theories exist to explain this phenomenon including the prevailing hypothesis that MTX mediates its anti-inflammatory effects in RA through the indirect modulation of extracellular adenosine concentrations [6,7,8]. MTX is an analogue of folic acid and acts as a potent inhibitor of the enzyme dihydrofolate reductase (DHFR), resulting in the systemic depletion of biologically active forms of folate [9]. The major biologically active form of folate found in plasma is 5-methyltetrahydrofolate (5mTHF), whereas RBCs contain both 5mTHF and 5,10-methenyltetrahydrofolate (CH=THF). In addition to its potent effects on DHFR, MTX is also metabolized intracellularly to form a series of polyglutamated metabolites (MTX-Glu_n_), which can directly inhibit various folate-dependent enzymes. Numerous studies in the human autoimmune arthritis disease population have found that levels of these metabolites are associated with the efficacy of MTX [10]. These metabolites contain up to seven glutamate residues and have a distinct pharmacological profile from MTX [1,11]. However, it is unclear if these metabolites represent the pharmacologically active form of MTX or serve as a pharmacokinetic biomarker of MTX exposure.

One approach to study the development and progression of RA, as well as therapeutic response, is the collagen-induced arthritis (CIA) mouse model [12]. As the most commonly used animal model to study autoimmune arthritis, the CIA mouse model produces a systemic inflammatory response that resembles the physiological and pathogenic characteristics of RA and has been demonstrated to be responsive to disease modifying therapies, such as MTX [13,14,15,16]. Previous work by our group has demonstrated that MTX therapy in the CIA mouse model results in a dose-dependent reduction in disease activity and is associated with a significant depletion of circulating folates despite relatively minimal metabolism of MTX to MTX-Glu_n_ [1]. This observed reduction in folate levels with minimal formation of MTX-Glu_n_ suggests that the pharmacological inhibition of DHFR by MTX may be critical to the efficacy of MTX in this model and supports folate level monitoring as a pharmacodynamic biomarker of MTX response in autoimmune arthritis.

Recognizing that analysis of MTX disposition and anti-folate activity represents a targeted and relatively biased approach to biomarker identification, in this study we expand our approach to include semitargeted global metabolomic profiling to identify metabolic changes associated with disease induction and response to MTX. Metabolomics represents the comprehensive analysis of all small-molecule metabolites in a biological specimen at a given time point [17,18]. This powerful tool is emerging as a strategy for precision medicine and biomarker discovery, allowing for rapid analysis of hundreds to thousands of low molecular weight compounds and providing insight into global metabolic changes associated with disease and in response to drug therapy [18,19,20,21]. As a result, this work seeks to use the CIA mouse model to verify previously identified metabolic markers of MTX efficacy in the CIA mouse model (i.e., folates) and to identify novel potential therapeutic biomarkers and biochemical pathways associated with MTX efficacy using semitargeted global metabolomic profiling.

## 2. Results

### 2.1. Efficacy of MTX in the CIA Mouse Model

MTX efficacy in autoimmune arthritis was evaluated in the CIA mouse model by measuring disease activity score (DAS) and paw volumes (PV) as approximations of disease activity. DAS was followed throughout the 54-day duration of the study and PV was measured at baseline and upon completion of the study. Mice (*n* = 30) were first randomly assigned to one of three evenly distributed groups, including healthy control mice (con), CIA disease mice (dis), and CIA disease mice treated with MTX (dis + MTX). A total of 27 mice remained at the completion of the study, with three mice (two from the disease group and one from the dis + MTX group) expiring during, or shortly after, their intradermal tail injections during disease induction booster dosing (presumably due to inadvertent injection of the collagen emulsion into the tail vein).

**Figure 1:**
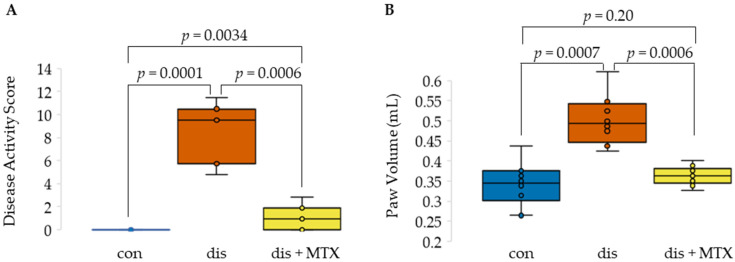
Effects of MTX on disease activity in the CIA mouse model. Box and whisker plots displaying (**A**) disease activity scores and (**B**) paw volumes comparing healthy control mice (con; *n* = 10), CIA induced disease mice (dis; *n* = 8), and CIA mice treated with MTX (dis + MTX; *n* = 9).

A standard CIA protocol was followed for induction of autoimmune arthritis, resulting in a disease induction rate of 100%, with disease defined as a DAS of 1 or greater [1,15]. Statistically significant increases in DAS and PV were observed in the CIA disease mice compared to the healthy control mice (Figure 1). For the disease mice treated with MTX, subcutaneous MTX was first administered on day 14 upon the first observable signs of disease activity at a dose of 20 mg/kg and repeated once per week for the duration of the study. Mice in the disease group received corresponding subcutaneous injections of saline (i.e., placebo) once per week.

At the completion of the study, both DAS and PV were significantly lower in MTX-treated mice compared to disease mice, with a total of six (67%) mice in the dis + MTX group having measurable disease activity (Figure 1). No measurable disease activity was observed in the healthy control group, with the difference in PV between healthy control and dis + MTX groups not reaching statistical significance. Effects of MTX on disease activity in this CIA mouse model were in line with those observed in previous studies [1,13,14,15].

### 2.2. MTX Disposition and Effect on Systemic Folates in the CIA Mouse Model

**Figure 2:**
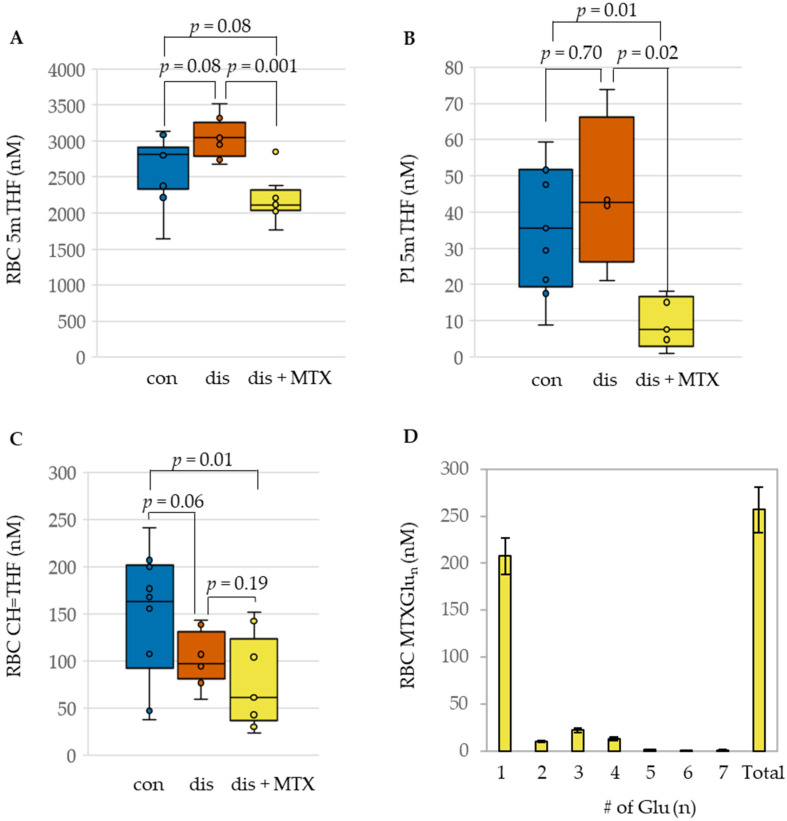
Influence of MTX treatment on folate concentrations and RBC disposition of MTX in the CIA mouse model. Comparison of (**A**) RBC 5mTHF, (**B**) Plasma 5mTHF, (**C**) RBC CH=THF, and (**D**) polyglutamate distribution of RBC MTX in healthy control (con; blue; *n* = 10), CIA disease mice (dis; orange; *n* = 8), and CIA disease mice treated with MTX (dis + MTX; yellow; *n* = 9).

Having demonstrated the effectiveness of MTX at reducing arthritis disease activity in the CIA mouse model, measurement of MTX disposition and its anti-folate effects were undertaken to evaluate the relationship of these markers with MTX efficacy. Concentrations of MTX and its polyglutamate metabolites were measured in RBCs, along with the major folate isoforms present in plasma (i.e., 5mTHF) and RBCs (i.e., 5mTHF and CH=THF) (Figure 2).

Upon induction of disease, modest impacts on folates were observed when compared to the control mice. This included an increase in RBC 5mTHF levels (Figure 2A) and a corresponding reduction in RBC CH=THF levels; these differences didn’t reach statistical significance (Figure 2C). Compared to the disease mice, treatment with MTX was associated with a statistically significant reduction in concentrations of 5mTHF in both plasma and RBC. Although a decrease was also observed in RBC CH=THF concentrations, it was not found to be statistically significant. In addition to the observed changes in folate levels in RBC and plasma following MTX therapy, MTX disposition in RBCs was also quantified (Figure 2D). Approximately 80% of total MTX in RBCs was represented by the parent form of MTX, providing additional evidence of a relative lack of intracellular polyglutamation of MTX in the CIA mouse model.

### 2.3. Association of MTX Efficacy with MTX Disposition and Anti-Folate Activity

**Figure 3:**
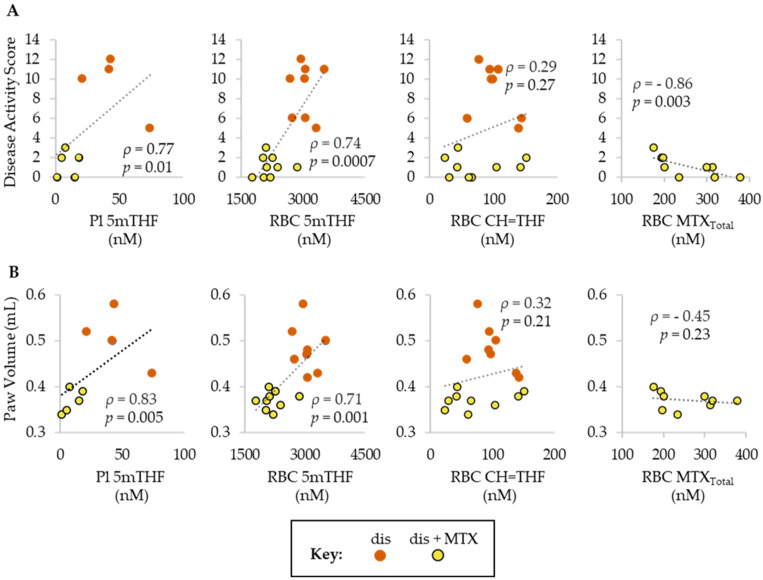
Spearman’s correlations of (**A**) DAS and (**B**) PV with folate (5mTHF and CH=THF) and MTX (MTX_Total_) levels in plasma and/or RBCs. Groups include, dis (orange; *n* = 8) and dis + MTX (yellow; *n* = 9).

After quantifying the disposition of MTX and its effect on systemic folates in the CIA mouse model, we sought to determine the relationship of these molecular markers with the efficacy of MTX. Folate levels in disease mice and disease mice treated with MTX were subjected to Spearman’s regression analysis to define the correlation between folate levels and measures of disease activity (i.e., DAS, PV) (Figure 3). Both plasma and RBC 5mTHF levels were found to be directly associated with DAS and PV, whereas RBC CH=THF levels failed to demonstrate a significant association with either DAS or PV. MTX disposition represented by the total RBC concentration of MTX and its polyglutamated metabolites (RBC MTX_Total_) was only measurable in mice treated with MTX, and was found to be inversely associated with DAS but failed to demonstrate a significant association with PV.

### 2.4. Plasma Metabolomic Changes Associated with CIA Disease Induction and the Effect of MTX

**Figure 4:**
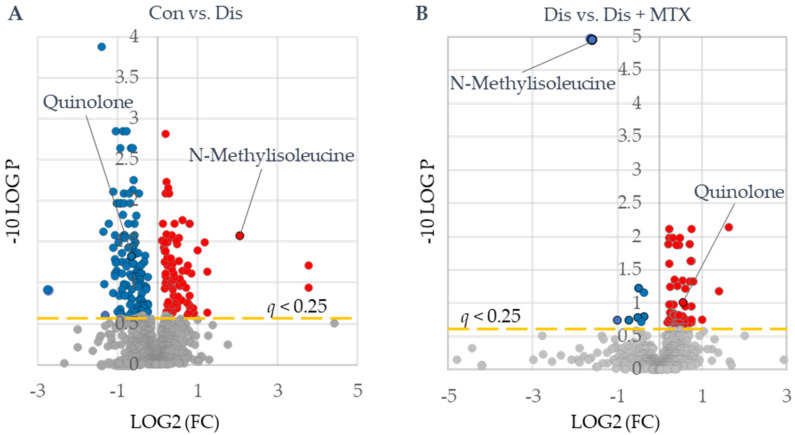
Identification of metabolites of interest associated with CIA disease induction and MTX therapy. Plasma metabolomics data were subjected to univariate analysis using MetaboAnalyst 5.0 providing volcano plots for (**A**) control (Con) (*n* = 10) vs. disease mice (Dis) (*n* = 8) and (**B**) disease mice (Dis) (*n* = 8) vs. disease mice treated with MTX (Dis + MTX) (*n* = 9). Red-colored metabolites were found to increase, and blue-colored metabolites were found to decrease (*q* < 0.25).

Following the assessment of the disposition of MTX and its effect on systemic folates in the CIA mouse model, plasma samples were analyzed by semitargeted global metabolomic profiling. Plasma metabolomic data included semiquantitative measurement of 1012 identified metabolites and included intermediates of primary metabolism, lipids, and biogenic amines. Metabolomic data were analyzed using an unpaired univariate analysis and are presented as volcano plots (Figure 4). These plots were used to identify compounds that significantly differed between disease and control mice (Figure 4A) and between disease mice and disease mice treated with MTX (Figure 4B). Metabolites that met an FDR-adjusted *p*-value (i.e., *q* value) threshold of less than 0.25 were regarded as metabolites of interest. Based on this threshold, a total of 210 metabolites were found to differ between CIA disease mice and healthy control mice (Table S1), with 125 metabolites found to decrease and 85 observed to increase. Similarly, a total of 79 metabolites were found to significantly differ between MTX-treated mice and the disease mice (Table S2), with 11 metabolites found to decrease and 67 observed to increase. Of the metabolites identified, only two metabolites, N-methylisoleucine (NMI) and quinolone, were found to be altered upon disease induction and subsequently returned towards healthy control levels following treatment with MTX.

Differences in measured metabolites were then subjected to chemometric enrichment analysis conducted using ChemRICH, an open-source software, to produce nonoverlapping chemical cluster mapping (Figure S1). While changes in the metabolome were observed, particularly within metabolites associated with fatty acid/lipid metabolism, no metabolite clusters were found to display a correction towards healthy control levels following MTX therapy in disease mice. One noteworthy observation was a significant reduction in pyrimidine nucleosides in disease mice treated with MTX when compared to the disease control mice (*p* = 0.03).

In order to further access the relationships of metabolites of interest identified via univariate analysis, a metabolic network map was built in MetaMapp and visualized using Cytoscape v3.8.4 (Institute for Systems Biology, Seattle, WA, USA) to highlight the differences in the plasma metabolomes between the comparison groups (Figure S2). Metabolomes were plotted onto network maps and compounds were organized based on metabolic pathways, and labeled based on chemical similarity. While metabolic network mapping revealed changes in the overall metabolome, including metabolites within nucleosides, ceramides, sphingomyelins, phosphatidyl-lipids, fatty acids, and cholesterol esters that were associated with disease induction and MTX treatment, the only partial correction observed was in fatty acids where initial decreases trended back up towards the healthy control levels. Taken together, these analyses reveal that while CIA induction and MTX therapy in mice result in significant changes to the metabolome, the observed changes do not represent a shift towards healthy control levels.

### 2.5. Association of Metabolomic Markers with MTX Efficacy

**Figure 5:**
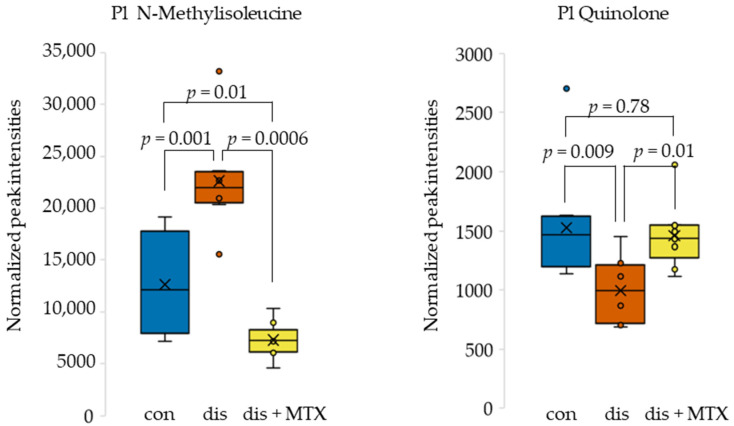
Box and whisker plots of plasma metabolite levels for metabolites identified as altered in disease mice and corrected in disease mice receiving MTX therapy. Control (blue; *n* = 10); disease (orange; *n* = 8); and dis + MTX (yellow; *n* = 9).

The previously identified metabolites of interest, NMI and quinolone, showed altered levels in disease and displayed a normalization towards healthy control levels following MTX therapy. The metabolite levels for NMI and quinolone in control, disease, and MTX-treated disease mice were plotted as box and whisker plots (Figure 5). NMI levels were found to be elevated in disease mice and were markedly reduced with MTX treatment. In contrast, quinolone levels were reduced in disease mice and increased towards control levels with MTX treatment. Mean levels of NMI were three-fold lower in MTX treated mice compared to disease mice, whereas mean levels of quinolone were 31% higher in MTX treated mice compared to disease mice.

**Figure 6:**
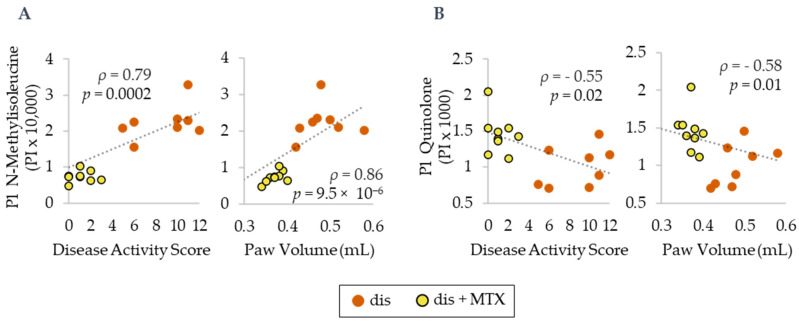
Spearman’s correlations for plasma metabolite levels in normalized peak intensity (PI) of (**A**) N-methylisoleucine and (**B**) quinolone with PV and DAS. Dis (orange; *n* = 8) and dis + MTX (yellow; *n* = 9).

In order to assess the relationship between MTX efficacy and the identified plasma metabolomic markers, NMI and quinoline levels were analyzed by Spearman’s regression analysis to define their correlation with DAS and PV (Figure 6). Plasma NMI levels were found to be directly associated with both DAS and PV. In contrast, plasma quinolone levels were found to be inversely associated with both DAS and PV. NMI was found to have a strong correlation, and quinolone was found to have a moderate correlation with disease activity measures.

### 2.6. Comparison of Folates and Metabolomic Markers as Biomarkers of MTX Response

**Figure 7:**
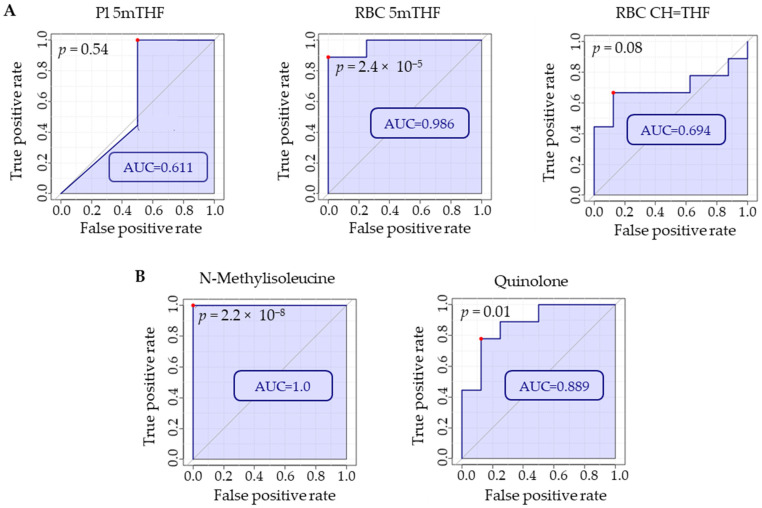
Using MetaboAnalyst 5.0, ROC curve analyses were conducted on (**A**) folate levels in plasma and RBCs and (**B**) plasma metabolites identified by semitargeted global metabolomic profiling (i.e., NMI and quinolone) to compare which markers were most discriminatory with regard to MTX treatment. Red dots on each plot indicate the optimal cut-off based on specificity and sensitivity. Comparison is based on metabolite levels in RBC or plasma for disease mice (*n* = 8) and disease mice treated with MTX (*n* = 9).

After MTX efficacy was found to be associated with the metabolomic markers NMI and quinolone, receiver-operating characteristic (ROC) curve analysis was conducted in MetaboAnalyst 5.0 to determine how they compare to folates as biomarkers of MTX treatment. ROC curve analysis is useful for determining the accuracy of a diagnostic test or biomarker by plotting the false positive rate (i.e., 1-specificity) versus the true positive rate (i.e., sensitivity) resulting in an area under the curve (AUC). Our data support NMI and quinolone as strongly discriminatory between MTX-treated and disease mice when comparing the AUC for each ROC curve (Figure 7). Both NMI and quinolone were able to reliably discriminate between MTX-treated and disease mice. Among the measures of folate, RBC 5mTHF was found to strongly discriminate based on MTX treatment, while Pl 5mTHF and RBC CH=THF did not reach statistical significance and had much lower AUCs compared to the other biomarkers.

The MTX-treated mice were further stratified based on response to MTX treatment to evaluate whether any of the metabolic markers were related to response within this group. Specifically, using both DAS and PV measurements, mice below the median for each of the measurements within the group were considered responsive, and those at or above the median were considered nonresponsive for the analysis. By DAS, mice considered responsive to MTX (*n* = 3) all had a DAS of 0, and mice considered nonresponsive (*n* = 6) had a median [IQR] DAS of 1.5 [1, 2.25]. None of the identified plasma or RBC markers were found to discriminate based on DAS response (*p* > 0.05). Mice with a cumulative paw volume less than 0.37 mL were considered responders (*n* = 3) and those with a PV of 0.37 mL or greater were considered nonresponders (*n* = 6) with a median [IQR] PV of 0.35 [0.34, 0.6] and 0.38 (0.37, 0.39). Of the plasma and RBC markers compared between these groups, only NMI was found to significantly differ. Stratification of disease mice treated with MTX based on response by PV found that responsive mice had significantly lower median [IQR] plasma NMI levels compared to mice determined to be nonresponsive (*p* = 0.04).

## 3. Discussion

This study evaluated molecular biomarkers and biochemical pathways associated with MTX response through the use of pharmacologically targeted and semitargeted global metabolomic analyses in the CIA mouse model. Our CIA protocol induced robust and consistent autoimmune arthritis and was highly responsive to treatment with MTX. MTX treatment was found to be strongly associated with the reduction of two independent measures of arthritis disease activity, DAS and PV. These reductions in disease activity were associated with reduced levels of 5mTHF in RBCs and plasma following MTX treatment. Contrary to previous findings in human trials, we found only a low level of accumulation of MTX polyglutamates in RBCs [10]. Semitargeted metabolomic data were processed via unpaired univariate analysis and revealed that two plasma metabolites, NMI and quinolone, were significantly altered (*q* < 0.25) after disease induction and were both corrected following MTX treatment. Changes in NMI and quinolone levels were significantly correlated with reductions in disease activity measures and discriminated based on MTX treatment, as evidenced by ROC curve analysis. Our findings support RBC 5mTHF levels and plasma levels of NMI and quinolone as potential molecular biomarkers of MTX response and provide support for the hypothesis that the anti-folate effect of MTX is associated with its efficacy in autoimmune arthritis. However, it must be noted that the association of these metabolites with the efficacy of MTX in this mouse model of autoimmune arthritis should not be interpreted as mechanistically causative of drug response, and may represent biochemical effects of MTX therapy that correlate with response to therapy but may not represent the mechanistic basis for drug action. Further mechanism-based studies are needed to fully understand the molecular basis for MTX efficacy in autoimmune arthritis, and the role of its anti-folate effects.

To best inform clinical practice decisions, accurately modeling RA is crucial for generating applicable translational research. Using this rationale, the CIA mouse model was selected to model RA because it produces consistent systemic inflammation that resembles RA and has been demonstrated to be responsive to MTX [1,13,14,15]. In clinical practice, administration of subcutaneous weekly MTX is considered to be a superior alternative to oral administration for patients who are able to adhere to weekly injections [1,22,23]. This regimen aims to reduce the risk of MTX-related toxicities associated with an extended dosing interval and the avoidance of variable oral bioavailability related to the saturable transporter mediated absorption across the gastrointestinal lumen [1,24,25,26]. In this study, MTX was dosed at 20 mg/kg and administered subcutaneously based on previous dose-escalation studies done by this lab [1]. In spite of the pharmacokinetic differences between the two routes of administration, observations made in this study are expected to similarly apply to the use of oral MTX [27].

While MTX has been a cornerstone therapy against autoimmune diseases for decades, the mechanism associated with its efficacy is still debated [6,7,8]. Some have suggested that MTX efficacy is independent of the folate depleting effects of MTX and is primarily dependent on the extracellular accumulation of adenosine secondary to inhibition of de novo purine biosynthesis [7,28]. In this CIA model, RBC 5mTHF levels were found to decrease nearly 33% with MTX treatment, and while CH=THF levels were found to decrease as well, this was not found to be statistically significant. A strong positive correlation was observed in RBC and plasma levels of 5mTHF with measures of disease activity. This observation of reduced folate levels being directly associated with MTX efficacy contradicts previous clinical studies that found no changes in circulating folate levels in patients treated with MTX [1,29,30]. In fact, these previous observations served as the justification that pharmacologic activity of MTX in autoimmune arthritis is independent of its anti-folate effects. Despite this, our findings corroborate with previous studies from our group, which also found an approximate 30% reduction in circulating folate concentrations following MTX treatment in the CIA mouse model [1]. The decreased levels of 5mTHF could be explained by MTX inhibition of DHFR, resulting in a depleted pool of bioactive folates, resulting in a state of effective folate deficiency [6,30]. If MTX exerts its pharmacologic effects via depletion of folates, it may be logical to question whether folate supplementation would impair its efficacy. Folic acid supplementation has become a standard treatment strategy with the aim of trying to reduce potential toxicities associated with MTX treatment [31]. In response to this potential question, our previous analysis of folate levels in JIA patients demonstrated an approximately 30% reductions in systemic levels of biologically active folate levels despite supplementation with folic acid [32]. Overall, our data demonstrate a correlation between MTX efficacy and reductions in folates, as well as the ability of RBC 5mTHF to act as a marker of MTX response; however, more studies are needed to fully elucidate these relationships in the autoimmune arthritis patient population.

Having found that depletion of circulating folates is associated with the efficacy of MTX in our CIA mouse model, MTX disposition in RBCs was also quantified. In this study, roughly 20% of MTX in RBCs was represented by MTX-Glu_n_. Despite a low level of MTX polyglutamation, efficacy with MTX was still achieved. The finding of low levels of MTX polyglutamate formation in the CIA mouse model was initially paradoxical due to the known importance of MTX polyglutamates to the pharmacologic activity of MTX [1,33,34]. However, this finding agrees with our previous reports of approximately 10 to 25% of tissue MTX to exist as a polyglutamate in the DBA/1J mice. This is in contrast to the high level of polyglutamation typically seen in patients, with approximately 80% of RBC folate as MTX-Glu_n_ [35]. While a low level of polyglutamation was observed in our CIA mouse model, total RBC concentrations of MTX and its metabolites displayed a statistically significant inverse relationship with DAS but not PV. This finding along with our previous findings on the anti-folate effect of MTX suggest inhibition of DHFR by MTX, resulting in depletion of the biologically active folate pool, is an important component of MTX efficacy in the CIA mouse model.

In addition to the folate and MTX disposition analysis, a semitargeted plasma metabolomic analysis was conducted to identify changes in biological pathways associated with MTX efficacy and disease induction. To better understand the effects that MTX exerts on the metabolome, it is necessary to first understand the impact that autoimmune arthritis has on the metabolome. Significant changes were found in the metabolome when comparing control mice to disease mice. A majority of these changes occurred in lipid metabolism, including increases in cholesterol esters (*p* = 0.001), phosphatidylcholines (*p* = 1.2 × 10^−5^), sphingomyelins (*p* = 1.9 × 10^−4^) and decreases in unsaturated triglycerides (*p* = 2.2 × 10^−20^) and ceramides (*p* = 0.03) (Figure S1). These changes are unsurprising based on the established relationship between autoimmune disorders and dyslipidemia, likely related to the established role of lipids in inflammation and adaptive immunity [36,37]. Increases in systemic inflammation have been associated with the stimulation of lipolysis and may explain the decrease in plasma triglycerides observed in the disease mice [38,39,40]. Unlike previous works, these changes in the metabolome associated with disease induction were not corrected following MTX treatment [41]. This finding, paired with the lack of polyglutamation of MTX, may indicate that MTX has a discrete pharmacodynamic and pharmacokinetic profile in the CIA mouse model when compared to humans.

Although MTX did not correct some of the presumed pathologic metabolic changes associated with disease in our dataset, it still displayed a significant effect on the metabolome. Some changes associated with MTX treatment were predicable based on its known mechanism of action; for example, MTX therapy was associated with decreases in pyrimidine nucleosides. Through inhibition of DHFR, MTX inhibits the synthesis of the purines and pyrimidines that are necessary for nucleic acid synthesis [8,28]. MTX was also associated with changes in metabolites not directly associated with its presumed pharmacological effects, with significant increases in NMI levels observed upon disease induction, and a three-fold reduction observed with MTX treatment. Plasma levels of NMI were strongly correlated with disease activity by both DAS and PV. NMI is a methylated product of the essential amino acid isoleucine; however, reports on its physiological functions are scarce. Previous works have identified this metabolite as a natural product of bacterial metabolism; however, it is not known if this metabolite is the product of an exogenous source (e.g., gut microbial metabolism) or is generated endogenously by N-methyltransferases [42]. One possible explanation for the correction of NMI could be the effect of MTX on one-carbon metabolism resulting in a reduction in methyltransferase activity. The biologically active forms of folate serve as methyl donors and, as a result, are key regulators of one-carbon metabolism; thus, a depletion of folates would be expected to impact the methylation of various biomolecules, including amino acids [43,44,45,46,47]. In addition to the correlation of plasma NMI levels with measures of disease activity, ROC curve analysis demonstrated that NMI outperformed folates in discriminating between disease animals and those treated with MTX. Further, among disease mice treated with MTX stratified based on response, lower plasma NMI levels were associated with response based on PV. Together, these data provide further evidence of the potential role of NMI as a biomarker of MTX response.

The second identified metabolite of interest, quinolone, displayed levels that were reduced post disease induction and returned towards healthy control levels after MTX treatment. Prior metabolomic studies have not reported on this metabolite. However, alkylated quinolone metabolites were first discovered to be produced by *Pseudomonas aeruginosa* and display potent antimicrobial properties, which have led to the development of fluroquinolone antibiotics [48]. RA has been associated with dysbiosis of gut microbiota, and recent evidence suggests the gut microbiome plays a role in modulating host immune function and response to MTX therapy [49,50]. In addition, evidence exists of MTX treatment being able to potentially shift a ‘diseased’ microbiome toward a ‘healthy’ microbiome [49]. Furthermore, microbiota possess extensive potential to metabolize xenobiotic compounds (e.g., pharmaceuticals) and can have considerable implications for drug stability and activity [50,51,52,53]. Thus, it is crucial to elucidate how MTX and other DMARD therapies can impact the microbiome and consequently influence treatment response and biomarker identification. It is notable that quinolone and NMI were unlikely to have been ingested from exogenous sources by the mice, and neither have been shown to be generated from endogenous processes in humans or mice; therefore, it is plausible given previous evidence that levels of these metabolites were altered due to changes in the microbiome from CIA induction and MTX therapy. However, it must be noted that other metabolic intermediates commonly associated with alteration in gut microbial metabolism, such as indoles, secondary bile acids, and fatty acids, were not found to be associated with disease induction and MTX response. Due to this evidence, and our lack of understanding of the source of plasma quinolone and NMI, further research is necessary on the gut metabolome in order to confirm the origins of these biomolecules to properly assess them as biomarkers of MTX efficacy. As a result, a major limitation to this work was the absence of an analysis of the gut microbiota composition in these mice and the collection of data related to their dietary intake, as these are important contributors to the gut microbial ecosystem and may account for the effects of the microbiome on the plasma metabolome observed here. Together, our data support the potential use of RBC 5mTHF, quinolone and NMI as biomarkers of MTX response based on their correlation with measures of disease activity and their ability to discriminate between MTX-treated and disease mice by ROC curve analysis.

## 4. Materials and Methods

### 4.1. Animals

Male DBA/1J mice were purchased at 6 to 8 weeks old from Jackson Laboratory (Bar Harbor, ME, USA) and kept under pathogen-free conditions at an Association and Accreditation of Laboratory Animal Care approved Animal Care Unit at The University of Kansas. All experimental procedures were conducted in accordance with the Guide for the Care and Use of Laboratory Animals as adopted and declared by the U.S. National Institutes of Health, and were conducted under a protocol approved by the Institutional Animal Care and Use Committee at The University of Kansas.

### 4.2. Disease Induction and Treatment

Following a one-week acclimation period, mice (7 to 9 weeks old) undergoing the disease induction protocol were given intradermal tail injections of a commercially prepared collagen/CFA emulsion containing 1 mg/mL chicken type II collagen and 2 mg/mL killed mycobacterium tuberculosis H37Ra (Hooke Laboratories, Lawrence, MA, USA) at experimental day 0. Afterwards they received a subsequent intradermal tail booster injection on day 19 of collagen/IFA emulsion containing 1 mg/mL chicken type II collagen [15].

The study included healthy control mice (*n* = 10), mice undergoing disease induction (*n* = 8), mice undergoing the disease induction protocol treated receiving once weekly subcutaneous doses of MTX (*n* = 9) at 20 mg/kg. MTX dosing was initiated concurrently in all mice following the earliest observable signs of disease as determined by disease activity scoring. This resulted in a total of six weekly doses administered over the 54-day experimental period.

Mice were fed with dry normal chow pellets *ad libitum*. When mice in the disease groups developed autoimmune arthritis, normal chow was substituted with normal chow soaked in water that was replaced daily. Mice were closely monitored throughout the study for humane endpoints. Mice were monitored two to three times weekly throughout the duration of the study for disease activity scoring, and paw volume measurements were taken once at the beginning and following completion of the study. On experimental day 54, all mice were euthanized by CO_2_ asphyxiation, after which plasma and RBC samples were collected for analysis.

### 4.3. Disease Activity Scoring

Disease activity scores were measured as an assessment of autoimmune arthritis in all four limbs using an established scoring system [15]. In short, disease severity score between 0 and 4 was determined by two investigators for each limb by visual inspection. If no evidence of erythema and swelling of the limb a score of 0 assigned, a score of 1 corresponded to erythema and swelling isolated to a single digit, a score of 2 was assigned for erythema and swelling in more than 1 digit or mild swelling of the entire paw, a score of 3 was given for erythema and swelling of the entire paw, and a score of 4 specified severe swelling of the entire paw or limb ankylosis. Disease activity scores were calculated as sum of all four limbs (maximum score of 16) and was individually tracked for each animal over the duration of the study.

### 4.4. Paw Volume Measurements

Disease severity was also monitored routinely through measuring paw volume using limb water volume displacement [54]. Briefly, paw volumes were determined by submerging each paw into a water bath and measuring the resulting volume of water displaced with a syringe. Hind and front paws were submerged up to and including the ankle and elbow joints, respectively. Paw volume measurements were collected on day 0 and day 54 and were reported as the sum of the total water volume displaced by all four paws for each animal.

### 4.5. Methotrexate and Folate Analysis

Blood samples were obtained by cardiac puncture following euthanization at experimental day 54 and were collected into potassium EDTA-containing tubes. Separation of blood into red blood cells (RBCs) and plasma fractions was accomplished by centrifugation at 1600× *g* for 10 min at room temperature. The resulting RBC pellet was washed once by resuspension in PBS at room temperature and pelleted by centrifugation at 1600 *g* for 10 min at room temperature. RBC and plasma samples were stored at −80 °C prior to analysis. RBC and plasma samples were analyzed using an established UPLC-MS/MS method to quantify the content of MTX and its polyglutamate metabolites containing up to a total of seven glutamic acid residues [1,31,32]. RBC and plasma 5mTHF levels and RBC CH=THF levels were determined using previously established UPLC-MS/MS assays [55]. Samples were normalized and reported in nmole of analyte per liter of either packed RBC or plasma volume.

### 4.6. Metabolomics Analysis

Following euthanization of the mice, plasma samples were submitted to the National Institutes of Health (NIH) West Coast Metabolomics Center at the University of California, Davis (Davis, CA, USA) for semitargeted analysis. Sample preparation conducted by West Coast Metabolomics Center included the use of biphasic liquid-liquid extraction using three independent standardized analytical methods to measure intermediates of primary metabolism, biogenic amines, and lipids. Afterwards, curated raw peak intensity data were obtained, and a standardized normalization procedure was performed. The normalization procedure involved taking the ratio of the sum of all peak heights for all metabolites for each sample (mTIC) to the average mTIC for all samples and repeating the process for all metabolites. The observed peak heights for each metabolite were then divided by the normalization ratio, providing the normalized peak height intensity. Duplicate metabolites detected from different analytical methods were combined by mean normalization and averaged to preserve equal weighing between methods. The resulting data were uploaded into MetaboAnalyst 5.0 where normalization through logarithmic transformation with Pareto scaling was applied. Volcano plots were generated to identify fold changes and differentiating metabolites.

### 4.7. Enrichment Analysis

Using fold-change and *p*-values generated from MetaboAnalyst, metabolites were analyzed via chemical and metabolic networks. Visualization of chemometric and biochemical network maps were built with MetaMapp. Network maps used Kyoto Encyclopedia of Genes and Genomes (KEGG) identifications and Tanimoto substructure similarity coefficients to organize metabolites based upon chemical similarity. The processed data were then visualized in Cytoscape 3.8.4 (Institute for Systems Biology, Seattle, WA, USA). Enrichment analysis was performed utilizing the open-source software Chemical Similarity Enrichment Analysis for Metabolomics (ChemRICH), accessed on 30 June 2021. ChemRICH utilizes chemical ontologies and structural similarities to generate non-overlapping sets of identified metabolites. This method does not rely on background databases or biochemical pathways. PubChem chemical information database was used to find predicted octanol:water partition coefficient (xlogP) of metabolites (https://pubchem.ncbi.nlm.nih.gov/, accessed on 30 June 2021).

### 4.8. Statistical Analysis

A significance threshold was determined *a priori,* and a false discovery rate adjusted *p* value (*q* value) less than 0.25 was used in both metabolomic analysis. Chemometric analysis was accomplished using ChemRICH, with significant metabolites having a *q* value of less than 0.05. Statistical analyses, including linear regression, significance testing and Spearman’s rank correlation, were gathered via JMP Pro 15 (SAS Institute, Cary, NC, USA).

## 5. Conclusions

In conclusion, the key finding in this study is that MTX treatment in the CIA mouse model results in a reduction in arthritis disease activity and is associated with the effect of MTX on circulating folate concentrations as well as the newly identified plasma metabolomic markers, NMI and quinolone. Together, these results support NMI and quinolone as potential biomarkers of MTX therapy in autoimmune arthritis, and indicate that RBC 5mTHF levels may represent an accessible pharmacodynamic biomarker of MTX efficacy in the treatment of autoimmune arthritis. One limitation to metabolomic methods is that they are hypothesis-generating in nature and fail to provide mechanistic details on the observed trends. Rather, they can only identify such associations and additional studies are needed to confirm the association of RBC 5mTHF, as well as plasma quinolone and NMI, with MTX efficacy in autoimmune arthritis and to understand the mechanistic basis for this association.

## Data Availability

The datasets generated and analyzed for this study have been submitted to Metabolomics Workbench database (https://www.metabolomicsworkbench.org, submitted on 6 December 2021 under Data Track ID 2895).

## Supplementary Materials

The following are available online at https://www.mdpi.com/article/10.3390/metabo12010024/s1. Figure S1: Chemometric network mapping and metabolomic enrichment data was analyzed using ChemRICH software which produced nonoverlapping chemical group classifications that mapped 1012 metabolites to 68 nonoverlapping chemical classes. (A) Metabolomic changes associated with CIA disease induction where compared to healthy control mice. Of these 68 classes differentiating control and CIA mice, 9 were found to be statistically significant (*p* < 0.05). (B) Metabolomic differences associated with disease mice treated with MTX, compared to untreated CIA disease mice. Of these classes differentiating CIA mice and MTX treated CIA mice, 5 were found to be statistically significant (*p* < 0.05). Each group of chemicals was assessed based on lipophilicity and fractional directional change. Node size of each cluster was directly proportional to the negative log of the *p* value for each class, Figure S2: Metabolic network maps were built with MetaMapp 2020 and visualized using Cytoscape 3.8.4. The metabolomic networks were organized into clusters using the community cluster tool and labeled by metabolite class. Red colored nodes represent metabolites that were found to significantly increase, and blue nodes represent metabolites that were found to be significantly decreased. Node size was directly proportional to measured fold-change. (A) induction of CIA vs control (B) MTX treatment in CIA mice vs untreated CIA mice. Table S1: Data corresponding from volcano plots showing metabolites that were significantly different (*q* < 0.05) between control (con) and disease (dis) mice, Table S2: Data corresponding from volcano plots showing metabolites that were significantly different (*q* < 0.05) between disease (dis) and disease mice treated with MTX (dis+MTX).
