# Supplementary material for: Methotrexate Disposition, Anti-Folate Activity, and Metabolomic Profiling to Identify Molecular Markers of Disease Activity and Drug Response in the Collagen-Induced Arthritis Mouse Model
Source: Metabolites. 2021 Dec 28;12(1):24. doi: 10.3390/metabo12010024 (PMC8780148; doi:10.3390/metabo12010024)
Supplement: Supplementary file 1 [file metabolites-12-00024-s001.zip › metabolites-1522348-supplementary.pdf]

metabolites MDPI

Supplemental Materials:

Table S1. Data corresponding from volcano plots showing metabolites that were significantly different ($q < 0.05$) between control (con) and disease (dis) mice.

Con v. Dis			
**Compound**	**FC**	***q* Value**	**Raw *p* Value**
PI 36:1	0.37	0.0019	3.11×10^{-6}
CE 16:1	1.74	0.0019	7.11×10^{-6}
PI 36:2	0.48	0.0019	7.28×10^{-6}
PI 38:2	0.52	0.0019	8.98×10^{-6}
Trimethylammonium	0.58	0.0019	9.43×10^{-6}
PI 35:2; PI 17:1-18:1;	0.52	0.0019	1.11×10^{-6}
TAG 66:18	2.34	0.0037	2.53×10^{-5}
CE 22:6	1.91	0.0058	4.94×10^{-5}
PI 34:1	0.54	0.0058	5.19×10^{-5}
4-Trimethylammoniobutanoic acid	0.62	0.0065	6.44×10^{-5}
PI 36:3	0.65	0.0065	7.02×10^{-5}
PI 34:2	0.65	0.0072	8.51×10^{-5}
TAG 62:13	1.83	0.010	0.00014
PE-Cer d40:1	1.71	0.010	0.00014
PI 38:6	0.73	0.013	0.00020
PE 40:7	1.48	0.015	0.00025
TAG 53:2	0.69	0.015	0.00027
N-Methylisoleucine	1.88	0.015	0.00027
PI 35:1	0.60	0.015	0.00029
SM d40:2	1.32	0.015	0.00030
PI 37:2	0.51	0.018	0.00038
TAG 51:1	0.66	0.024	0.00053
SM d38:1	1.31	0.024	0.00055
TAG 60:12 A	1.74	0.024	0.00059
TAG 60:12 B	1.72	0.024	0.00060
PI 35:2	0.62	0.024	0.00063
Phenylacetylglycine	1.84	0.027	0.00073
TAG 52:6	0.59	0.029	0.00088
SM d39:1	1.19	0.029	0.00091
PI 36:2	0.66	0.029	0.00093
TAG 49:1	0.66	0.029	0.00093
LPE 38:6	0.59	0.029	0.00094
ethanolamine	1.43	0.029	0.0010
SM d40:1	1.33	0.029	0.0010
TAG 51:2	0.63	0.029	0.0010
PC 42:10	1.33	0.029	0.0010
TAG 62:14	1.74	0.029	0.0011
SM d42:5	1.20	0.029	0.0011
PC 40:2e	0.54	0.029	0.0011
Sclareolide	0.53	0.030	0.0012
Cer-NP t40:0	0.53	0.033	0.0014

TAG 60:11	1.47	0.033	0.0014
PC 40:7	1.27	0.033	0.0014
PI 32:0	0.55	0.035	0.0015
TAG 49:2	0.55	0.038	0.0017
Cer-NP t41:0	0.46	0.038	0.0017
PI 38:5	1.33	0.040	0.0019
LPE 34:0	0.55	0.045	0.0021
PC p-42:4	0.79	0.046	0.0022
TAG 51:3	0.61	0.046	0.0023
lyxose	0.42	0.049	0.0025
pseudo uridine	1.40	0.049	0.0026
TAG 53:3	0.66	0.049	0.0026
.alpha.,.beta.-Thujone	0.56	0.049	0.0026
PE 34:2e	0.60	0.049	0.0027

Table S2. Data corresponding from volcano plots showing metabolites that were significantly different ($q < 0.05$) between disease (dis) and disease mice treated with MTX (dis+MTX).

**Dis v. Dis+MTX**			
**Compound**	**FC**	***q* Value**	**Raw *p* Value**
N,N-Diethyl-2-aminoethanol	0.32	$1.46x10^{-6}$	$1.45x10^{-9}$
N-Methylisoleucine	0.33	$1.12x10^{-5}$	$2.21x10^{-8}$
PI 40:6	1.51	0.0071	$2.10x10^{-5}$
PI 40:7	1.69	0.0078	$3.09x10^{-5}$
PI 38:6	1.39	0.0090	$4.45x10^{-5}$
PC 37:5	1.60	0.010	$6.55x10^{-5}$
PC 38:5	1.30	0.010	$6.80x10^{-5}$
PC 42:10	1.49	0.010	$8.09x10^{-5}$
PC 40:7	1.32	0.011	0.00010
PC p-42:4; or PC o-42:5	1.32	0.012	0.00012
PC 40:8	1.28	0.021	0.00023
PC 39:4	1.40	0.024	0.00029
TAG 54:3e	2.01	0.035	0.00045
PC 39:5	1.31	0.035	0.00048
LPC 20:5	1.40	0.039	0.00058
DG 36:4	0.70	0.040	0.00064
PC 38:2	1.25	0.040	0.00067
PC 37:4	1.28	0.042	0.00075
PC 38:6	1.15	0.044	0.00083
PI 38:6	1.46	0.045	0.00088

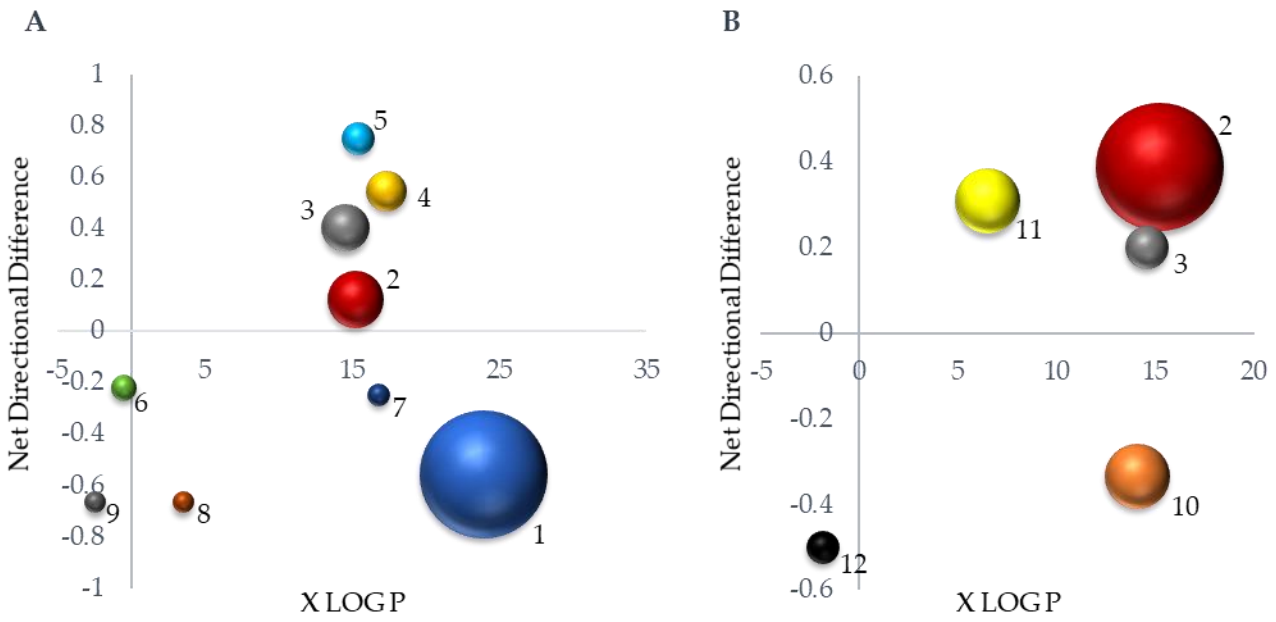

1. Unsaturated Triglycerides
2. **Unsaturated Phosphatidylcholines**
3. **Sphingomyelins**
4. Cholesterol Esters
5. Galactosylceramides
6. Dicarboxylic Acids
7. Unsaturated Ceramides
8. Terpenes
9. Glucuronates
10. Diglyceride
11. Unsaturated Lysophosphatidylcholine
12. Pyrimidine Nucleosides

Figure S1. Chemometric network mapping and metabolomic enrichment data was analyzed using ChemRICH software which produced nonoverlapping chemical group classifications that mapped 1012 metabolites to 68 nonoverlapping chemical classes. (**A**) Metabolomic changes associated with CIA disease induction where compared to healthy control mice. Of these 68 classes differentiating control and CIA mice, 9 were found to be statistically significant ($p < 0.05$). (**B**) Metabolomic differences associated with disease mice treated with MTX, compared to untreated CIA disease mice. Of these classes differentiating CIA mice and MTX treated CIA mice, 5 were found to be statistically significant ($p < 0.05$). Each group of chemicals was assessed based on lipophilicity and fractional directional change. Node size of each cluster was directly proportional to the negative log of the *p* value for each class.

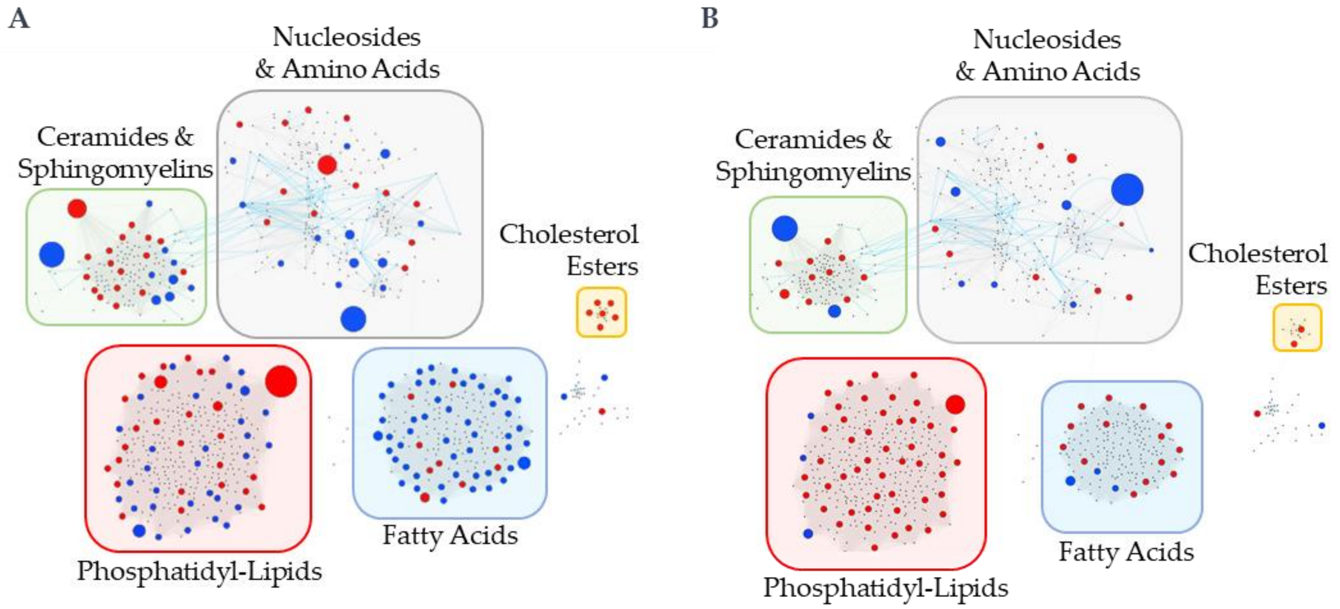

Figure S2. Metabolic network maps were built with MetaMapp 2020 and visualized using Cytoscape 3.8.4. The metabolomic networks were organized into clusters using the community cluster tool and labeled by metabolite class. Red colored nodes represent metabolites that were found to significantly increase, and blue nodes represent metabolites that were found to be significantly decreased. Node size was directly proportional to measured fold-change. (**A**) induction of CIA vs control (**B**) MTX treatment in CIA mice vs untreated CIA mice.
